# A Wider Scope of Analysis on 38% Silver Diamine Fluoride

**DOI:** 10.7759/cureus.44010

**Published:** 2023-08-23

**Authors:** Haifa AlKhodier, Ghadeer Molla, Nouf AlAjaji, Nuha A Alkanhal, Mona AlSaykhan

**Affiliations:** 1 Pediatric Dentistry, King Faisal Specialist Hospital and Research Center, Riyadh, SAU; 2 General Dentistry, King Faisal Specialist Hospital and Research Center, Riyadh, SAU

**Keywords:** number of applications, restoration, arrest, caries progression, silver diamine fluoride

## Abstract

This research was conducted to further support previous studies on the effectiveness of 38% silver diamine fluoride on caries arrest. In addition, the number of applications, time between subsequent applications, type of teeth (primary vs. permanent), final restoration following treatment, and the type of restoration, if any, were assessed.

It is a retrospective longitudinal study that was conducted using a chart review technique for which a waiver of informed consent was obtained. The research participants’ privacy was ensured and protected. All participants’ identities remained anonymous and confidential. Furthermore, consent was collected from participants' sitters during their appointments as a protocol prior to applying 38% silver diamine fluoride (SDF). The current research was reviewed and approved by the Research Ethics Committee at King Faisal Specialist Hospital and Research Center (KFSH&RC). All investigators abided by the rules and regulations of the Government of Saudi Arabia, KFSH&RC, and the Research Advisory Council.

A population of pediatric patients with primary and/or mixed dentition at a tertiary care hospital who were treated at the dental office between the period from March 2020 to December 2022 were recruited. A consecutive non-random sampling technique was used. The inclusion criteria include cavitated, asymptomatic, and teeth with no signs or symptoms of pulpal involvement. The sample size estimation was done by considering different percentages of success in arresting dental carries by using 38% SDF, with different levels of precision of 95% confidence intervals and with 0.05 level of significance.

A 95% success was assumed in arresting dental carries by using 38% SDF, with a precision of ±5%, and with α=0.05, the required teeth sample size was 101. Anticipating some incomplete information and documentation, the sample size may be dynamic. Nighty-seven teeth both primary and permanent were included in this study. Data were analyzed using IBM SPSS Statistical software version 26.0 (Armonk, NY: IBM Corp.) for Windows. Descriptive statistics (mean standard deviation, frequencies, and percentages) were used to describe the quantitative and categorical outcome variables. Pearson’s chi-square test and Fisher’s exact test were used to compare the proportion success of 38% SDF in relation to the characteristics of study subjects. The odds ratios were used to quantify the measure of association between the success and other categorical study variables. A binary multiple logistic regression was used to identify the independent variables associated with the success of 38% SDF in arresting dental caries. A p-value of <0.05 and 95% confidence intervals were used to report the statistical significance and precision of results.

Our current research concluded that different variables associated with 38% SDF, such as the number of applications and the time-lapse between applications, had no significant effect on the successful results of the application. Moreover, receiving a final restoration did not affect the success of the treatment when compared to treating with 38% SDF alone. Additionally, the type of final restoration had no impact on the prognosis. Further research using standardized protocols for study designs, detection criteria, outcomes, and statistical designs is needed to support findings and establish treatment guidelines.

## Introduction

Silver diamine fluoride (SDF) is an odorless liquid that possesses silver’s antibacterial benefit and fluoride’s hardening effect [1-3]. In the United States, it is considered to be an efficient tool in the management plan for every patient [4]. Over a 100-year period, silver-containing chemicals were effective as strong antimicrobial agents and fluoride was extensively studied for its efficacy against caries and its ability to successfully remineralize carious lesions [1,5]. SDF is used to desensitize caries-free lesions such as molar incisor hypoplasia (MIH) and to arrest active caries in other patients as well. The application protocol is fairly simple and non-invasive with discoloration being the most commonly reported disadvantage [2]. Temporary staining of the skin and gingiva has been reported as well [1]. A large population may benefit from using 38% SDF, such as special healthcare needs, elderly groups, high caries risk patients, persons with difficulty in accessing dental care, large cavities on multiple teeth which cannot be treated at once, and uncooperative pediatric patients as it requires a minimum level of cooperation [1,4]. Furthermore, it was successfully used to manage extensively decayed asymptomatic teeth [2]. This can be beneficial in situations where medical circumstances do not support dental extraction.

Its wide use is attributed to its effectiveness and efficiency in situations where performing a dental restoration seems to be challenging. It was first approved by the US Food and Drug Administration in 2014 for Dentin hypersensitivity [3]. Interestingly, its effectiveness is two times that of the fluoride varnish [5]. Selection criteria for teeth are paramount when using 38% SDF. For instance, teeth with spontaneous pain, or with deep caries reaching the pulp, or those that are inaccessible for application even with the use of orthodontic separators should not be treated with 38% SDF [4].

Previous studies have reported that the highest caries prevention fraction of fluoride varnish was 21.3%. On the contrary, the lowest caries prevention fraction of 38% SDF was 70.3% [5]. When applied once, 38% SDF has shown effectiveness between 50% and 90% [6-8]. Therefore, post-application follow-up is important to assess the need for subsequent applications when deemed necessary [9,10]. If a final restoration is not to be placed, two applications are recommended [4]. Most research was conducted on primary teeth with little emphasis on permanent dentition [1]. When using 38% SDF, case selection is paramount. Symptomatic teeth, extensively decayed teeth with pulpal involvement, and inaccessible surfaces may render such treatment to be unfavorable [4]. Prior to application, a clear explanation of risks and benefits must be demonstrated to patients/sitters. Informed consent is to be obtained. Coating with protecting gel is recommended to prevent Henna-looking temporary staining of the skin and soft tissue. Following that, a careful application following the American Academy of Pediatric Dentistry (AAPD) chair guide is recommended as follows: while caries excavation prior to application is not mandatory, gross debris must be eliminated [4]. Then, a protective barrier may be applied to the lips, skin, and gingival tissue around the lesion. Further, isolation with cotton rolls is a must. After that, drying the cavity with compressed air prior to applying SDF is recommended. Then, a bent microbrush with one drop of SDF is sufficient for an entire appointment. It is recommended to rub the cavity for at least one minute, when possible, prior to drying the excess material with gauze. Finally, the entire dentition can be treated with 5% sodium fluoride to further strengthen the prevention purpose [4]. A follow-up appointment is recommended after a period of two to four weeks to assess the need for a subsequent application [4]. Silver diamine fluoride has successfully proven to be a positive tool that should be included in each patient’s management plan [4]. In the past, different concentrations have been studied, such as 12% SDF and 38% SDF [6]. However, a tendency towards a concentration of 38% was observed due to its higher success rates [6,8]. Furthermore, the number of applications per year was an area of interest as well [6]. Most research recommended a biannual application to achieve prolonged caries arrest [6,8]. Regardless of the treatment option, SDF is considered to be a low-cost, simple, and non-invasive tool for arresting caries [6].

## Materials and methods

This is a retrospective longitudinal study that was conducted using a chart review technique for which a waiver of informed consent was obtained. The research participants’ privacy was ensured and protected. All participants’ identities remained anonymous and confidential. Furthermore, consent was collected from participants' sitters during their appointments as a protocol prior to applying 38% SDF. The current research was reviewed and approved by the Research Ethics Committee at King Faisal Specialist Hospital and Research Center (KFSH&RC). All investigators abided by the rules and regulations of the Government of Saudi Arabia, KFSH&RC, and the Research Advisory Council.

Pediatric patients at a tertiary care hospital who were treated at the dental office between the period from March 2020 to December 2022 were recruited. A consecutive non-random sampling technique was used. The sample size estimation was done by considering different percentages of success in arresting dental carries by using 38% SDF, with different levels of precision of 95% confidence intervals, and with 0.05 level of significance. A 95% success was assumed in arresting dental carries by using 38% SDF, with a precision of ±5%, with α=0.05, and the required teeth sample size was 101. Anticipating the presence of incomplete information and documentation, the sample size may be adjusted dynamically.

Patients who were treated in the dental office were approached with a previously usual protocol for SDF treatment. The nature of treatment and the decision-making process were discussed with patients' sitters. Risks vs. benefits were discussed and verbal consent was collected and documented in patients' charts. After this, the AAPD chair guide for the SDF application was adopted in our research as follows: (1) skin, lips, and gum were coated with a protective layer of petroleum jelly (caution was taken not to apply the protective layer on the caveated lesions). (2) Mouth prop was used during the procedure to keep the mouth open. Then, cotton rolls were placed along with saliva ejectors to optimize isolation. (3) Cavities were cleaned from gross debris and compressed air was applied for 2 seconds to dry the cavities. (4) One drop of SDF was used for every five lesions. Then, the cavity was rubbed with SDF for 60-90 seconds when possible. (5) Care was taken to apply SDF only on caveated lesions and white spots/demineralization spots were avoided to avoid inadvertent staining. (6) Excess material was removed with gauze. (7) The remaining dentition was treated with 5% sodium fluoride. Furthermore, the teeth that were previously treated with SDF received a covering coat of 5% NaF at the end. (8) The sitter was instructed to prevent the patient from eating or drinking for 30 minutes. Following that, patients were seen on follow-up appointments for either subsequent applications or final restorations. The follow-up appointments were placed based on patients' medical status and ability to commute to the hospital, especially for those who were out of town.

This retrospective study included a total number of 97 teeth. The inclusion criteria were as follows: cavitated, asymptomatic, and with no signs or symptoms of pulpal involvement. Data were analyzed using IBM SPSS Statistical software version 26.0 (Armonk, NY: IBM Corp.) for Windows. Descriptive statistics (mean standard deviation, frequencies, and percentages) were used to describe the quantitative and categorical outcome variables. Pearson’s chi-square test and Fisher’s exact test were used to compare the proportion success of 38% SDF in relation to the characteristics of study subjects. The odds ratios were used to quantify the measure of association between the success and other categorical study variables. A binary multiple logistic regression was used to identify the independent variables associated with the success of 38% SDF in arresting dental caries. A p-value of <0.05 and 95% confidence intervals were used to report the statistical significance and precision of results.

## Results

Data were analyzed using SPSS software version 26.0 (Armonk, NY: IBM Inc.). Descriptive statistics (mean, standard deviation, frequencies, and percentages) were used to describe the categorical and quantitative variables. In bivariate analysis, Fisher’s exact test was used to observe the association between the categorical study variables, i.e., type of teeth (primary and permanent) and restoration (yes and no), and outcome variable, i.e., success (high and fail). A p-value of ≤0.05 was used to report the statistical significance of results.

A total of 97 teeth were included in this study. The age range of all participants was between 2 and 13 years with a mean age of 5.88 years. Out of the 97 teeth, 83 were primary and the remaining 14 were permanent. About 89% of the teeth were restored with a success of 96.9%. The mean time since application and final restoration was 9.65 months. The number of applications was used from one to three, where 40.2% of teeth had one application, 51.5% had two, and 8.3% with three applications. The time duration between applications was one to three months in 87.6% of teeth and the remaining was three to six months and more than six months. The type of restoration was observed to be composite resin in 33%, 12.4% stainless-steel crowns (SSC), pulpotomy+SSC in 11.3%, Glass Ionomer Cement (GIC) in 9.3%, and extraction in 9.3% (Table 1).

**Table 1:** Elements studied when 38% silver diamine fluoride (SDF) was applied.

Considered elements	Mean±SD (no. %)
Age of subjects (in years)	5.88 (2.7)
Time since application and final restoration (in months)	9.65 (10.9)
Primary teeth included	83 (85.6)
Permanent teeth included	14 (14.4)
Number of all restored teeth following application	86 (88.7)
Number of all teeth left without final restoration	11 (11.3)
Number of composite resins as final restoration following application	33 (33.0)
Number of glass ionomer as final restoration following application	9 (9.3)
Number of stainless-steel crowns as final restoration following application	12 (12.4)
Number of stainless-steel crowns/pulpotomy as final restoration following application	11 (11.3)
Number of extracted teeth following application	9 (9.3)
Teeth that received three 38% SDF applications	8 (8.2)
Teeth that received two 38% SDF applications	50 (51.5)
Teeth that received one 38% SDF application	39 (40.2)
1-3 months between applications	85 (87.6)
3-6 months between applications	4 (4.1)
More than 6 months between applications	8 (8.2)
Successful results (absence of signs and symptoms)	94 (96.9)
Questionable results (such as abscess development)	3 (3.1)

The absence of signs and symptoms following application was recorded. Out of 83 primary teeth, 96.4% demonstrated successful results and all 14 permanent teeth showed successful outcomes as well. There was no statistically significant difference in the success of 38% silver diamine fluoride when used on primary or permanent teeth (p=0.470). Following 38% SDF application, 86 teeth were restored. Of these, 96.5% demonstrated favorable outcomes. For the remaining 11 teeth, no restoration was placed and the success rate was 100%. Thus, placing a restoration following 38% SDF application vs. not placing one showed no statistically significant difference (p=0.529) (Table 2). The success rate did not show any statistically significant differences based on other variables such as the type of restoration, the number of applications, and the time between applications.

**Table 2:** Association between 38% silver diamine fluoride treatment variables and treatment success.

Variables	High success	Fail	p-Value
Primary teeth	80 (96.4)	3 (3.6)	0.470
Permanent teeth	14 (100.0)	0 (0)	
Restored	83 (96.5)	3 (3.5)	0.529
Non-restored	11 (100.0)	0 (0)	-
Composite resin	33 (100)	0	
Glass ionomer	9 (100)	0	
Stainless-steel crown	12 (100)	0	
Stainless-steel crown/pulpotomy	9 (81.8)	2 (18.2)	
Extracted	8 (88.9)	1 (11.1)	
Others	11 (100)	0	
Three 38% SDF applications	6 (75.0)	2 (25.0)	
Two 38% SDF applications	50 (100.0)	0	
One 38% SDF application	38 (97.4)	1 (2.6)	
1-3 months between applications	84 (98.8)	1 (1.2)	
3-6 months between applications	4 (100.0)	0 (0)	
More than 6 months between applications	6 (75.0)	2 (25.0)	

## Discussion

Silver diamine fluoride (SDF) proved to be a successful, non-invasive, and cost-effective tool in arresting caries [11-14]. In addition, it proved to be a useful option when treatment under general anesthesia is not feasible [11]. In 2017, the AAPD recommended the use of 38% SDF as a treatment option for caries in pediatric, young adults, and special healthcare needs populations [15].

The current study was conducted at a tertiary care hospital at which most of the treated patients are considered to be American Society of Anesthesiology (ASA) III and ASA IV patients with severely compromising medical conditions and rare syndromes. Out of the 97 teeth (primary and permanent) included in this study, 94 demonstrated no signs or symptoms following 38% SDF application regardless of restoration availability. Only three primary teeth were extracted due to abscess development for which all subjects failed to show up earlier to their dental appointments with an average period of 15 months for not following up. All other extracted teeth have been removed due to inadequate tooth structure to hold permanent restoration, presence of a limited mouth opening due to syndromic condition, and presence of severe periodontitis rendering a tooth grade III mobility. In primary dentition, the effectiveness of 38% silver diamine fluoride has been studied and proven repetitively [16,17]. A one-year research studied the effect of 38% SDF on primary teeth with proximal lesions that are confined to dentin-enamel junction and found an 84% arresting rate of the lesions [17]. In permanent molars, the same effect was found on interproximal lesions in high-risk subjects [18]. Additionally, a meta-analysis of eight other studies has confirmed an 81% caries arrest rate on primary teeth [19]. One study has shown a reduction of the new active lesion percentage in both primary and permanent teeth following the application of 38% SDF [7]. Furthermore, a systematic review has proven the efficacy of 38% SDF on both primary and permanent molars with a higher cariostatic success when compared to different topical fluoride products [12]. However, some suggested further research to confirm its equal results on permanent molars [16].

Braga et al. studied the effect of 38% SDF on early lesions on 66 permanent molars. In the six-month follow-up, 38% SDF application had shown significant caries-arresting ability when compared to cross-tooth brushing and glass ionomer fissure sealants [20]. Also, research has shown that 38% SDF treatment performed very well on primary cuspids, permanent bicuspids, and molars [21]. Moreover, a superior effect of 38% SDF was found on permanent teeth when compared to sodium fluoride varnish [22]. The current study supported previous evidence as it showed a high success rate of using 38% silver diamine fluoride irrespective of the dentition age (primary or permanent).

In our research, 40.2% of teeth had one application, 51.5% had two, and 8.3% had three applications. The time duration between applications was one to three months in 87.6% of teeth. In 2018, an expert panel from the ADA made 11 recommendations based on a systematic review and meta-analysis regarding the use of 38% SDF, one of which was a biannual application protocol for both primary and permanent teeth [14,23]. Several studies have suggested that twice applications per year have rendered 38% SDF to be more effective [6]. Moreover, a single application of 38% SDF has been shown to be sufficient to inactivate the caries process for up to six months in primary teeth [24]. The AAPD chairside guide recommended a follow-up in two to four weeks with reapplication being indicated only in case of visible activity of the lesion. However, they recommended biannual application in cases where restorations will not be performed [4]. Furthermore, semiannual and single application of 38% SDF was sufficient with some results showing a 75% success rate [21,25]. Additionally, one to two applications have significantly remineralized carious dentin prior to placing glass ionomer restorations whereas subsequent applications demonstrated no superior effect [26]. The current study showed that the success rate was high and consistent irrespective of the number of applications and the time lapse between the applications. Furthermore, 89% of treated teeth have received restorations depending on the availability of tooth structure, cooperation level, caries risk assessment, and compliance to show for follow-up appointments with 33% being restored with composite resin restorations followed by 12.4% stainless-steel crowns (SSC), 11.3% pulpotomy/SSC, and 9.3% glass ionomer restorations.

Despite that, studies have shown that following 38% SDF treatment, a final restoration is not mandatory [27]. Clinical judgment, necessity of treatment, availability of equipment, and cooperation level need to be considered [27]. There seems to be insufficient evidence regarding the bond strength of adhesives and glass ionomer restoration to dentin [13]. For glass ionomer restorations, research showed no effect on glass ionomer restoration following 38% SDF treatment [13]. In fact, pretreatment with 38% SDF has eased the process and reduced the time required to place glass ionomer restorations in subsequent appointments [28]. This study showed a high success rate regardless of the type and time of placing the final restoration. While our results support previous ones for 38% SDF promising effectiveness, further research is needed to confirm the previous results and establish concrete guidelines for 38% SDF treatment protocol.

## Conclusions

Silver diamine fluoride at a concentration of 38% proves to be an effective management tool for arresting dental caries on primary and permanent dentition. Dentists and public health providers can benefit from SDF as it is a minimally invasive, low-cost, and efficient treatment. Our current research supported previous ones in studying different variables with 38% SDF, such as the number of applications and the time-lapse between applications. We concluded that they had no significant effect on the successful results of the application. Moreover, receiving a final restoration did not affect the success of the treatment when compared to treating with 38% SDF alone. Additionally, the type of final restoration had no impact on the prognosis. These findings can be useful when painless, fast, and simple treatment is required in situations where behavior is questionable or access to care may pose challenges to the patients medically or financially. We recommend a further search on 38% SDF treatment and inclusion of such an effective tool in each patient's dental care plan, even in situations where patients are adults with positive attitudes toward dental treatment. Such decision will assist in further caries prevention and structure destruction. Thus, minimize the need for possible expensive interventions (Root Canal Treatments {RCTs} and prosthodontic treatment) or irreversible interventions, such as extractions in the future.
